# The role of autophagy regulated by the PI3K/AKT/mTOR pathway and innate lymphoid cells in eosinophilic chronic sinusitis with nasal polyps

**DOI:** 10.1002/iid3.1310

**Published:** 2024-06-18

**Authors:** Jin‐Jing Zhuo, Chen Wang, Yi‐Long Kai, Ying‐Ying Xu, Ke‐Jia Cheng

**Affiliations:** ^1^ Department of Otolaryngology, The First Affiliated Hospital, School of Medicine Zhejiang University Hangzhou Zhejiang China

**Keywords:** autophagy, chronic sinusitis, eosinophil, phosphoinositide 3‐kinase, type 2 innate lymphoid cells

## Abstract

**Background:**

The PI3K/Akt/mTOR pathway and autophagy are important physiological processes. But their roles in eCRSwNP remains controversial.

**Methods:**

In this study, we used the eCRSwNP mouse model, PI3K/Akt/mTOR pathway inhibitors, and autophagy inhibitors and activators to investigate the regulatory effects of the PI3K/Akt/mTOR pathway on autophagy, and their effects on eosinophilic inflammation, and tissue remodeling. The role of ILC2s in eCRSwNP was also studied, and the relationship between ILC2s and autophagy was preliminarily determined.

**Results:**

Our results show that eosinophilic inflammation in eCRSwNP mice could be inhibited by promoting the autophagy; otherwise, eosinophilic inflammation could be promoted. Meanwhile, inhibition of the PI3K/Akt/mTOR pathway can further promote autophagy and inhibit eosinophilic inflammation. Meanwhile, inhibiting the PI3K/Akt/mTOR pathway and promoting autophagy can reduce the number of ILC2s and the severity of tissue remodeling in the nasal polyps of eCRSwNP mice.

**Conclusions:**

We conclude that the PI3K/Akt/mTOR pathway plays roles in eosinophilic inflammation and tissue remodeling of eCRSwNP, in part by regulating the level of autophagy. The downregulation of autophagy is a pathogenesis of eCRSwNP; therefore, the recovery of normal autophagy levels might be a new target for eCRSwNP therapy. Furthermore, autophagy might inhibit eosinophilic inflammation and tissue remodeling, in part by reducing the number of ILC2s.

## Graphical abstract

1. We conclude that the PI3K/Akt/mTOR pathway plays roles in eosinophilic inflammation and tissue remodeling of eCRSwNP, in part by regulating the level of autophagy. 2. For the first time, we speculate that autophagy can inhibit eosinophilic inflammation and tissue remodeling, in part by reducing the number of ILC2s. 3. We found that the downregulation of autophagy was a pathogenesis of eCRSwNP.

## 1 INTRODUCTION

Chronic sinusitis with nasal polyps (CRSwNP) is a common disease that seriously affects the quality of life of patients. It is classified into two subtypes according to the infiltrating inflammatory cells: eosinophilic (eCRSwNP) and noeosinophilic subtypes.^1^ The etiology of eCRSwNP is complex and its pathogenesis is unclear. *Staphylococcus aureus* play an important role in the pathogenesis of eCRSwNP, not only by *S. aureus* enterotoxin B, but also by leukocidin ED.^2^ It readily recurs after surgery, and tends to develop into refractory chronic sinusitis (CRS). Persistent eosinophilic inflammation and tissue remodeling are important reasons for the recurrence and refractoriness of eCRSwNP.^3^

Autophagy is a protein degradation pathway of eukaryotic cells that is dependent on lysosomes. It is an important physiological process for adapting to the environment and maintaining the stability of the internal environment. Autophagy can protect cells and tissues and has anti‐inflammatory effects, helping the body clear irritants and invading pathogens. However, abnormal autophagy has different roles in different diseases or different stages of the same disease. It plays important roles in tumor, infection, inflammation, tissue fibrosis, and neurodegenerative and autoimmune diseases.^4^ In asthma, autophagy can regulate eosinophilic inflammation and tissue remodeling by affecting fibroblast and eosinophil (EOS) apoptosis, EOS differentiation in bone marrow, and their secretion function.^5^ Recent studies have suggested that autophagy plays a role in eCRSwNP, but it remains controversial. Choi et al.^6^ found that autophagy defects promoted the development of eosinophilic CRS. On the contrary, Wang et al.^7^ showed that the level of autophagy in the nasal polyps of eCRSwNP patients was increased. The role of autophagy in eosinophilic inflammation and tissue remodeling of eCRSwNP remains unclear.

Autophagy is regulated by multiple signaling pathways, among which the phosphoinositide 3‐kinase/protein kinase B/mammalian target of rapamycin (PI3K/Akt/mTOR) pathway is particularly important. The PI3K/Akt/mTOR pathway widely exists in a variety of cells and is involved in regulating their growth, proliferation, migration, survival, and differentiation. It also plays roles in many physiological and pathological processes. Class Ⅲ PI3K and mTOR complex 1 (mTORC1) in the PI3K/Akt/mTOR pathway can regulate autophagy^8^; however, the PI3K/Akt/mTOR pathway has rarely been studied in eCRSwNP and its role remains unclear. Kim et al. found that PI3k‐110δ was increased in eCRSwNP.^9^

In this study, we used the eCRSwNP mouse model, PI3K/Akt/mTOR pathway inhibitors, and autophagy inhibitors and activators to investigate the regulatory effects of the PI3K/Akt/mTOR pathway on autophagy, and their effects on eosinophilic inflammation, and tissue remodeling. The role of type 2 innate lymphoid cells (ILC2s) in eCRSwNP was also studied, and the relationship between ILC2s and autophagy was preliminarily determined. The results of this study reveal the pathogenesis of eCRSwNP, providing a basis for the development of novel therapeutic targets.

## 2 MATERIALS AND METHODS

### 2.1 eCRSwNP mouse model and experimental groups

An eCRSwNP mouse model was established according to a previous study^10^ as follows. Briefly, 4‐week‐old wild‐type mice (C57BL/6 mice) were sensitized by intraperitoneal (IP) injection of ovalbumin (OVA) (25 μg OVA soluble in 300 μL phosphate buffered saline (PBS) and 2 mL aluminum hydroxide) at 0 and 5 days. One week after the second injection, 3% OVA was administered intranasally once a day for 1 week, and then three times a week for 12 weeks. In the last 8 weeks, medication was administered until sampling. At the same time, for the last 8 weeks, infusion of *Staphylococcus aureus* enterotoxin B was stimulated intranasally at 10 ng (soluble in 20 μL PBS) per time, once a week. OVA sensitization and stimulation induced the allergic mouse model. *S. aureus* enterotoxin B was added to induce nasal polyp formation, forming eCRSwNP mouse model. Nasal polyps, nasal lavage fluid, and peripheral blood samples were collected 24 h after the last stimulation. Seventy‐two C57BL/6 mice were divided into 12 groups with six mice in each group. Systemically (IP or intragastric injection) and locally administered (nasal drip) PBS (20 μL), rapamycin (3 mg/kg; MedChemExpress), chloroquine (CQ; 15 mg/kg; Leyan), LY294002 (0.3 mg/kg; MedChemExpress), 3‐methyl adenine (3‐MA; 15 mg/kg; MedChemExpress), and the PI3K inhibitor AS605240 (50 mg/kg; Sigma‐Aldrich) were used twice a week. AS605240 was the only drug administered intragastrically.

### 2.2 Automatic biochemical detection

Peripheral blood was collected into Vacutainer tubes. The total blood count was performed by the Hunter Area Pathology Service (Salamander Bay, NSW, Australia), using the Beckman Coulter LH Hematology Analyzer. The amount of EOS was measured.

### 2.3 Enzyme‐linked immunosorbent assay (ELISA)

The levels of interleukin 5 (IL‐5), eotaxin, and eosinophil cationic protein (ECP) (Thermo Fisher Scientific) in nasal lavage fluid were detected by the ELISA. In brief, the samples (40 μL) were incubated in 96‐well plates with the appropriate antibody (10 μL) for 30 min at 37°C. Wash buffer was added to each well for 30 s, and then gently tapped out. This procedure was performed five times. Next, 50 µL of horseradish peroxidase Conjugate Reagent was added to each well, and the plate was incubated and washed as before. Chromogen solution A and chromogen solution B (50 μL each) were added to each well, and the plates were gently mixed and incubated for 15 min at 37°C. Next, 50 µL of stop solution was added to each well, and the optical density (OD) at 450 nm was read within 15 min. A standard curve was generated to enable quantification.

### 2.4 Western blot assay

The protein expression of PI3K, phosphorylated PI3K (p‐PI3K), Akt, phosphorylated Akt (p‐Akt), mTOR, phosphorylated mTOR (p‐mTOR), Beclin‐1, and microtibule associated protein kinase light chain3‐II/Ⅰ (LC3Ⅱ/LC3Ⅰ) (Cell Signaling Technology) in nasal polyps was detected by Western blotting (WB). In brief, tissue was ground, followed by the addition of lysis solution, incubation on ice for 30 min, and centrifugation at 12,000 rpm for 15 min at 4°C. The BCA Protein Assay Kit was used to detect protein concentration. Proteins were resolved by sodium dodecyl sulfate‐polyacrylamide gel electrophoresis and electrotransferred to membranes. The membranes were blocked, and then incubated overnight at 4°C with primary antibody. The next day, the membranes were washed five times with TBST, and then incubated with secondary antibody at 37°C for 2 h. Then, the membrane was washed again five times with TBST. Proteins were visualized by enhanced chemiluminescence.

### 2.5 Flow cytometry

The ratio of ILC2s in nasal polyps was determined by flow cytometry (Beckman Coulter, cytoFLEX). Briefly, the cells were first sorted using side scatter and forward scatter clustering methods. Then CD3^−^ and CD45^+^ cells were sorted. Lin^−^ (CD1a^−^CD3^−^CD14^−^CD16^−^CD19^−^CD34^−^CD94^−^CD123^−^BDCA2^−^FcεR1α^−^TCRαβ^−^TCRγδ^−^) and CD127^+^ cells were screened using a pedigree marker antibody and CD127 antibody. CRTH2^+^ cells were finally selected (Abcam) (Thermo Fisher Scientific). Flowjo software was used for data analysis.

### 2.6 Cell staining and immunohistochemistry (IHC)

Hematoxylin and eosin staining (BASO) was used to observe EOS infiltration and the degree of epithelial cell injury in nasal polyps. As previously described for Masson's trichrome (MT) staining,^11^ blue collagen was used as a marker of extracellular matrix components (Solarbio). Briefly, the tissue was dewaxed and then stained with Weigert's iron hematoxylin for 10 min. Then the tissue was differentiated with acid ethanol for 5 s, washed with water, stained with MT solution, and washed again with water for 3 min. After staining with Ponceau S staining solution for 10 min, the tissue was washed with ammonium molybdophosphate solution for 1 min, stained with aniline blue for 1 min, and dehydrated with ethanol. This was followed by vitrification with dimethylbenzene and sealing with neutral gum. ImageJ (National Institutes of Health, Rockville, MD, USA) software was used to count the percentage of blue‐stained area in any three high‐power fields (HPFs).

Periodic acid–Schiff Alcian blue (PAS‐AB) staining (Solarbio) was used to identify goblet cells and mucus glands. Briefly, tissue was dewaxed, washed in distilled water for 2 min, and then stained with Alcian blue for 10 min. After washing three times with distilled water, the tissue was incubated with an oxidizing agent for 5 min, stained with PAS‐AB for 10 min, and flushed with water for 10 min. The nucleus was stained with hematoxylin for 2 min and washed with water. Then, the tissue was differentiated with acid ethanol for 5 s, washed with water, stained with Scott's solution, and washed again with water for 3 min. The final steps were dehydration in ethanol, vitrification by dimethylbenzene, and sealing with neutral gum. ImageJ software was used to count the percentage of positive cells in any three HPFs.

IHC was used to detect p‐Akt, p‐mTOR, Beclin‐1, and LC3II (Abcam) expression and distribution in nasal polyps. In brief, the tissue was dewaxed and washed, followed by incubation with 3% hydrogen peroxide at room temperature for 20 min to block endogenous peroxidase activity. Then, the tissue was washed again, incubated with 50 μL of goat serum at room temperature for 15 min, and then incubated with 50 μL of primary antibody (1:500) for 20 min. After washing three times with PBS, tissue was incubated with 50 μL of secondary antibody for 15 min. Then, the tissue was incubated with 50 μL of *Streptomyces* antibiotin protein‐peroxidase solution for 15 min. Next, the solution was removed and washed three times with PBS. Finally, tissue was stained with 100 μL of DAB solution followed by hematoxylin counterstaining. After dehydration in ethanol, vitrification by dimethylbenzene, and sealing, the tissue was observed by light microscopy. Integrated OD/area staining quantification was carried out with Image‐Pro Plus software (Media Cybernetics).

### 2.7 Transmission electron microscopy (TEM)

The formation of autophagosome in nasal polyps was observed by TEM. Briefly, tissues were fixed in 2.5% glutaraldehyde for 48 h, and then rinsed six times with PBS at 4°C, 30 min each. Then the tissue was fixed at 4°C in 1% osmium tetroxide for 2 h, followed by rinsing six times with PBS. The tissue was dehydrated in solutions of 50%, 70%, 90%, and 100% acetone, followed by incubation with 3 mL of pure acetone‐EPON812 embedding agent at 60°C for 24 h. The embedding agent was removed under a microscope. The embedded block was sliced into ultra‐thin sections with a thickness of 50 nm, and stained with toluidine blue. The slices were stored in a dry container for dyeing and embedded in paraffin. A few drops of sodium acetate dye were added to the paraffin sections. The edge of the carrier net was clamped and sliced side down so that the carrier net floated on the droplets. The petri dish was covered and dyed for 15 min. After dyeing, the petri dish was washed with double‐distilled water three times as soon as possible. Filter paper was used to absorb excess water, and placed in the petri dish to dry. The mesh was stained and cleaned with lead citrate in another petri dish with wax sheets in the same manner. After dyeing, the film was dried and observed by TEM.

### 2.8 Statistical methods

SPSS ver. 20.0 software (IBM Corp) was used to analyze the data. The data are expressed as the mean ± standard deviation, after they are verified as normal distribution by SPSS. The *t*‐test was used for intergroup comparison, and Pearson's test was used to detect the correlation between indicators. A *p* < .05 was considered statistically significant.

## 3 RESULTS

### 3.1 PI3K/Akt/mTOR pathway activity in eCRSwNP mice

**Figure 1:**
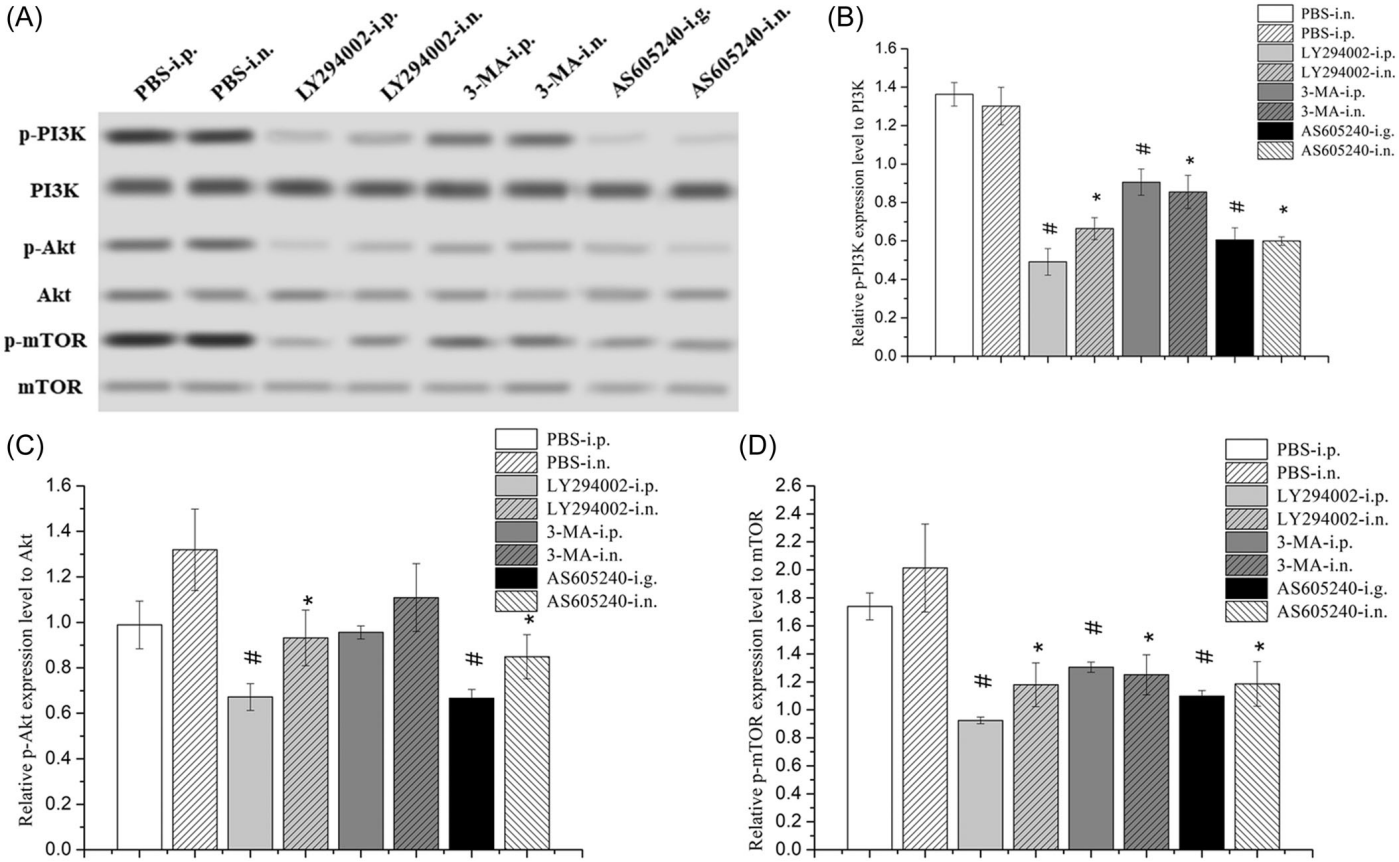
The protein expression of PI3K/Akt/mTOR pathway in nasal polyps detected by WB. (A) The WB bands of PI3K/Akt/mTOR pathway protein in nasal polyps of each group. (B) Comparison of p‐PI3K/PI3K protein expression in nasal polyps of each group. (C) Comparison of p‐Akt/Akt protein expression in nasal polyps of each group. (D) Comparison of p‐mTOR/mTOR protein expression in nasal polyps of each group. ^#^ *p* < .05 vs. PBS‐i.p. *p < .05 vs. PBS‐i.n. PBS, phosphate buffered saline; PI3K/Akt/mTOR, phosphoinositide 3‐kinase/protein kinase B/mammalian target of rapamycin; WB, Western blotting.

**Figure 2:**
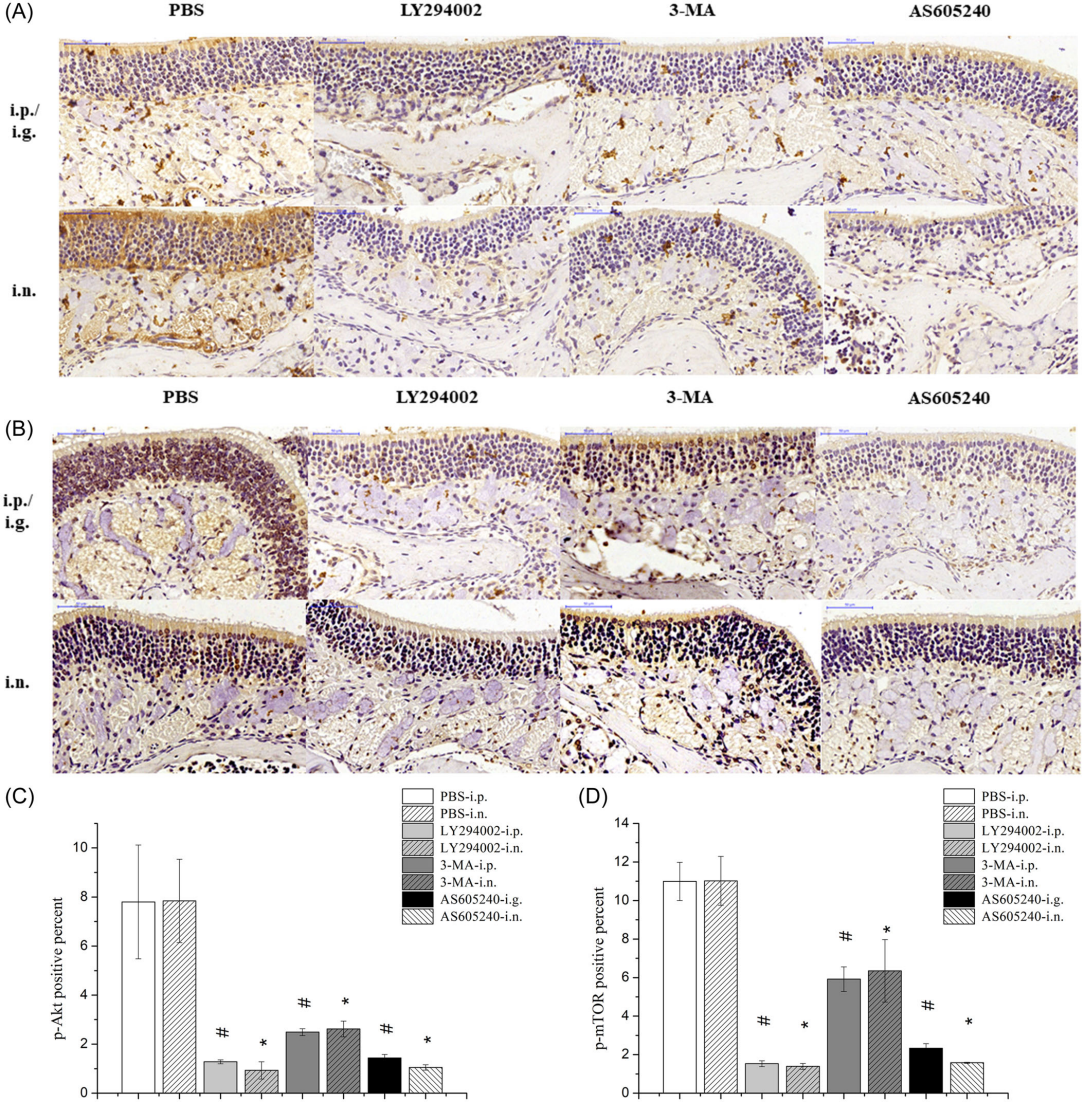
Expression of p‐Akt and p‐mTOR proteins in nasal polyps detected by IHC. (A) Expression of p‐Akt protein in nasal polyps of each group. (B) Expression of p‐mTOR protein in nasal polyps of each group. (C) Semi‐quantitative analysis and comparison of p‐Akt protein expression in nasal polyps of each group. (D) Semi‐quantitative analysis and comparison of p‐mTOR protein expression in nasal polyps of each group. ^#^ *p* < .05 vs. PBS‐i.p. *p < .05 vs. PBS‐i.n. IHC, immunohistochemistry; PBS, phosphate buffered saline; PI3K/Akt/mTOR, phosphoinositide 3‐kinase/protein kinase B/mammalian target of rapamycin.

WB revealed significantly decreased p‐PI3K/PI3K and p‐mTOR/mTOR protein expression ratios in nasal polyps after systemic and intranasal administration of LY294002, 3‐MA, and AS605240 compared with the control. Compared with the control, systemic and intranasal administration of LY294002 and AS605240 significantly decreased the p‐Akt/Akt protein expression ratio in nasal polyps. Moreover, the p‐Akt/Akt protein expression ratio was decreased, albeit non‐significantly, in the 3‐MA group (Figure 1A–D). IHC further showed that, compared with the control, systemic and intranasal administration of LY294002, 3‐MA, and AS605240 significantly reduced the expression of p‐Akt and p‐mTOR in nasal polyps (Figure 2A–D). Thus, systemic and intranasal administration of LY294002, 3‐MA, and AS605240 showed good inhibitory effects on the PI3K/Akt/mTOR pathway.

### 3.2 Levels of autophagy in eCRSwNP mice

**Figure 3:**
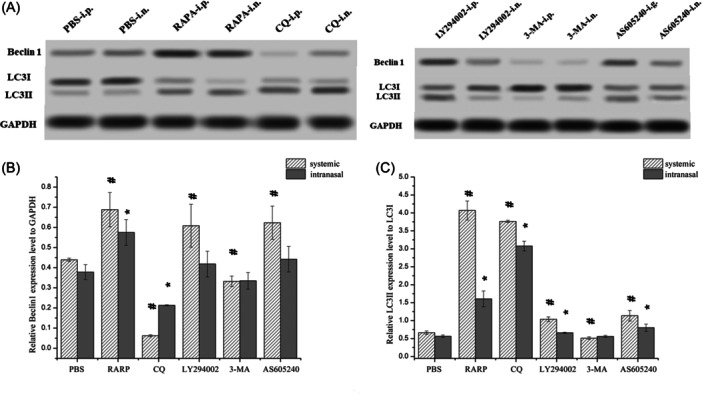
The protein expression of Beclin1 and LC3Ⅱ/LC3Ⅰ in nasal polyps detected by WB method. (A) WB results of Beclin1 and LC3Ⅱ/LC3Ⅰ in nasal polyps. (B) Comparison of Beclin1 protein expression in nasal polyps of each group. (C) Comparison of LC3Ⅱ/LC3Ⅰ protein expression in nasal polyps of each group. ^#^ *p* < .05 vs. PBS‐i.p. *p < .05 vs. PBS‐i.n. PBS, phosphate buffered saline; WB, Western blotting.

WB revealed significantly increased Beclin‐1 protein expression in nasal polyps after systemic application of rapamycin, LY294002, and AS605240 compared with the control. By contrast, systemic application of CQ and 3‐MA significantly reduced Beclin‐1 protein expression in nasal polyps. In addition, intranasal application of CQ significantly decreased Beclin‐1 protein expression in nasal polyps. Meanwhile, intranasal application of rapamycin significantly increased Beclin‐1 protein expression in nasal polyps compared with the control. Compared with the control, systemic, and intranasal rapamycin, CQ, LY294002, and AS605240 significantly increased LC3Ⅱ/LC3Ⅰ in nasal polyps. Finally, systemic application of 3‐MA significantly reduced LC3Ⅱ/LC3Ⅰ in nasal polyps (Figure 3A–C).

**Figure 4:**
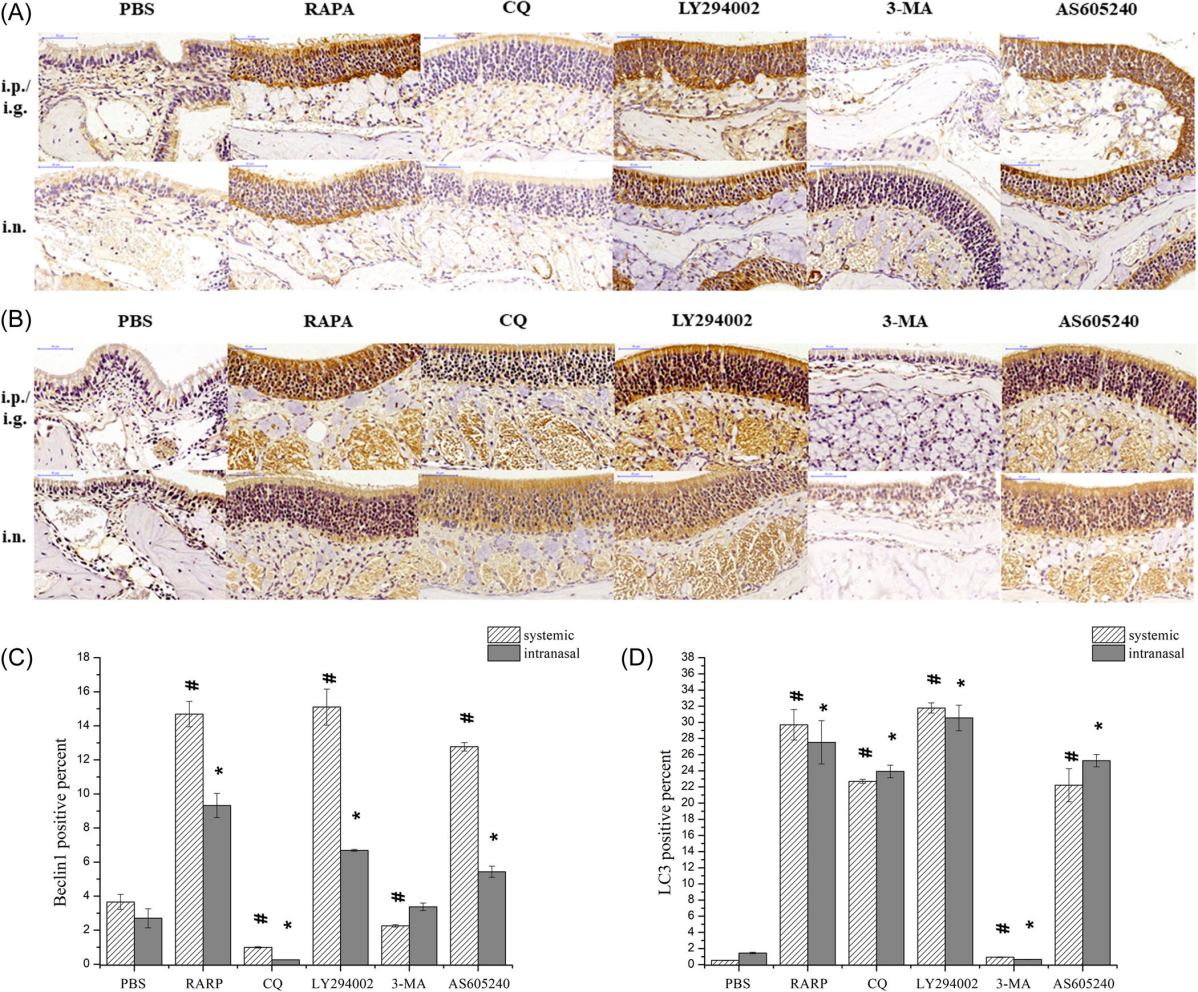
The expression of Beclin1 and LC3Ⅱ protein in nasal polyps detected by IHC. (A) The expression of Beclin1 protein in nasal polyps of each group. (B) The expression of LC3Ⅱ protein in nasal polyps of each group. (C) Comparison of Beclin1 protein expression in nasal polyps of each group. (D) Comparison of LC3Ⅱ protein expression in nasal polyps of each group. ^#^ *p* < .05 vs. PBS‐i.p. *p < .05 vs. PBS‐i.n. IHC, immunohistochemistry; PBS, phosphate buffered saline.

IHC revealed significantly increased Beclin‐1 protein expression in nasal polyps after both systemic and intranasal application of rapamycin, LY294002, and AS605240 compared with the control. By contrast, nasal application of CQ significantly decreased Beclin‐1 protein expression in nasal polyps (Figure 4A and 4C). Compared with the control, systemic and intranasal application of rapamycin, CQ, LY294002, and AS605240 significantly increased LC3Ⅱ protein expression in nasal polyps. Systemic and intranasal application of 3‐MA significantly reduced LC3Ⅱ protein expression in nasal polyps (Figure 4B and 4D).

**Figure 5:**
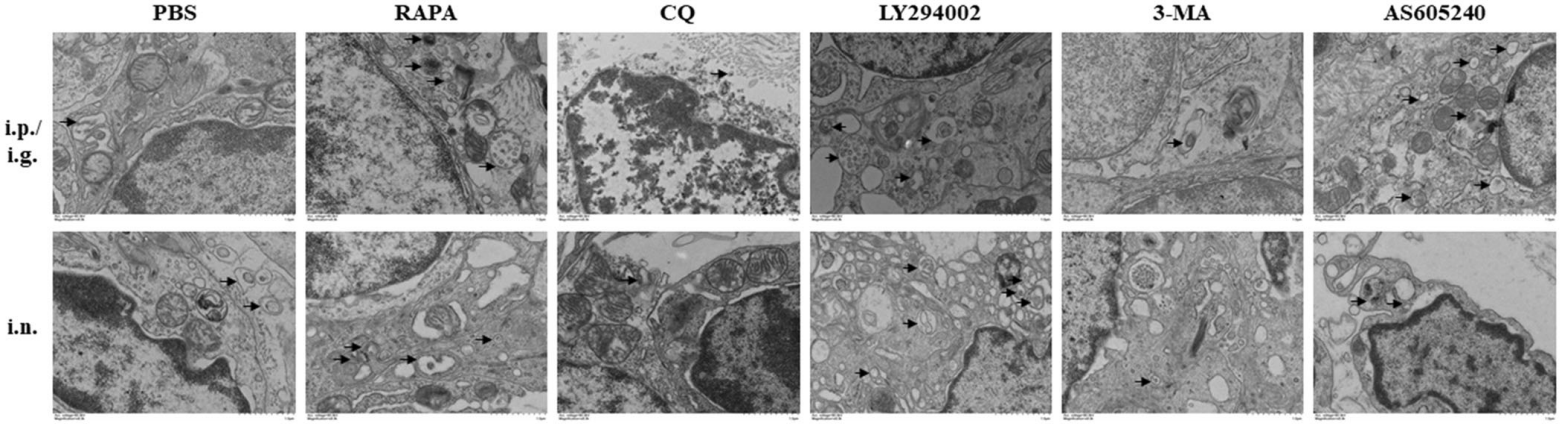
The formation of autophagosome in nasal polyps observed by TEM. The black arrows indicated the autophagosomes. TEM, transmission electron microscopy.

TEM revealed an increased number of autophagosomes in nasal polyps after systemic and intranasal application of rapamycin, LY294002, and AS605240, compared with the control. Meanwhile, CQ and 3‐MA reduced the number of autophagosomes in nasal polyps (Figure 5).

The above results indicate that rapamycin can promote autophagy in eCRSwNP. LY294002 and AS605240 can also promote autophagy by inhibiting the PI3K/Akt/mTOR pathway. CQ can inhibit autophagy and block LC3 degradation. 3‐MA has inhibitory effects on the PI3K/Akt/mTOR pathway; however, due to its blocking effect on Vps34, it has an overall inhibitory effect on autophagy. The effect of systemic administration of drugs was stronger than that of intranasal administration.

### 3.3 Eosinophilic inflammation in eCRSwNP mice

**Figure 6:**
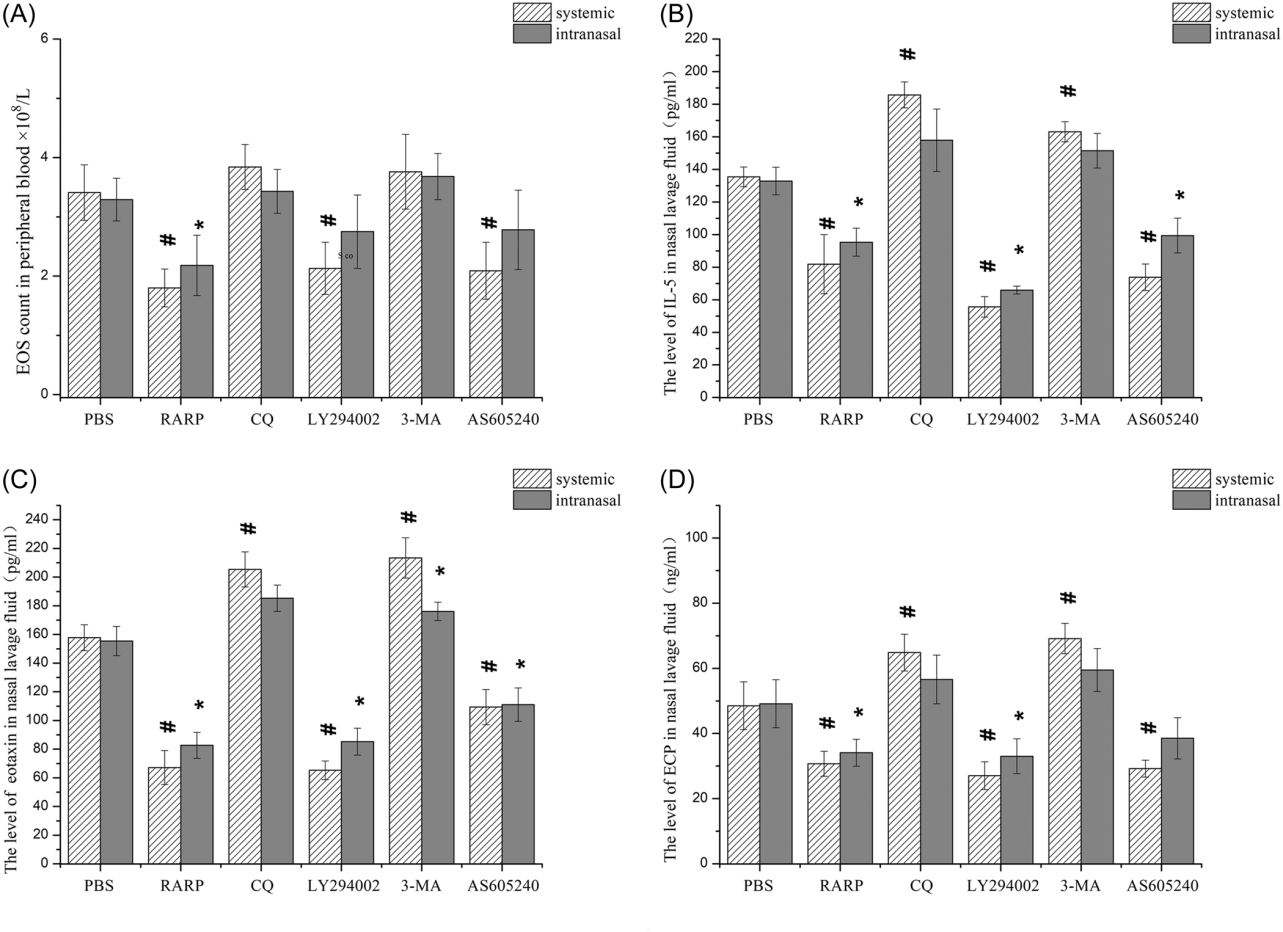
Peripheral blood EOS count and the levels of eosinophilic inflammatory factor in nasal lavage fluid in eCRSwNP mice. (A) EOS count in peripheral blood in each group. (B) The level of IL‐5 in nasal lavage fluid in each group. (C) The level of Eotaxin in nasal lavage fluid in each group. (D) The level of ECP in nasal lavage fluid in each group. ^#^ *p* < .05 vs. PBS‐i.p. *p < .05 vs. PBS‐i.n. EOS, eosinophil; PBS, phosphate buffered saline.

EOS count in peripheral blood revealed significantly reduced EOS after systemic application of rapamycin, LY294002, and AS605240 and intranasal application of rapamycin compared with the control (Figure 6A).

ELISA of nasal lavage fluid showed significantly decreased IL‐5, eotaxin, and ECP levels after systemic application of rapamycin, LY294002, and AS605240 compared with the control. Systemic application of CQ and 3‐MA significantly increased IL‐5, eotaxin, and ECP levels. However, the changes in IL‐5, eotaxin, and ECP levels were only partially significant during nasal administration (Figure 6B–D).

**Figure 7:**
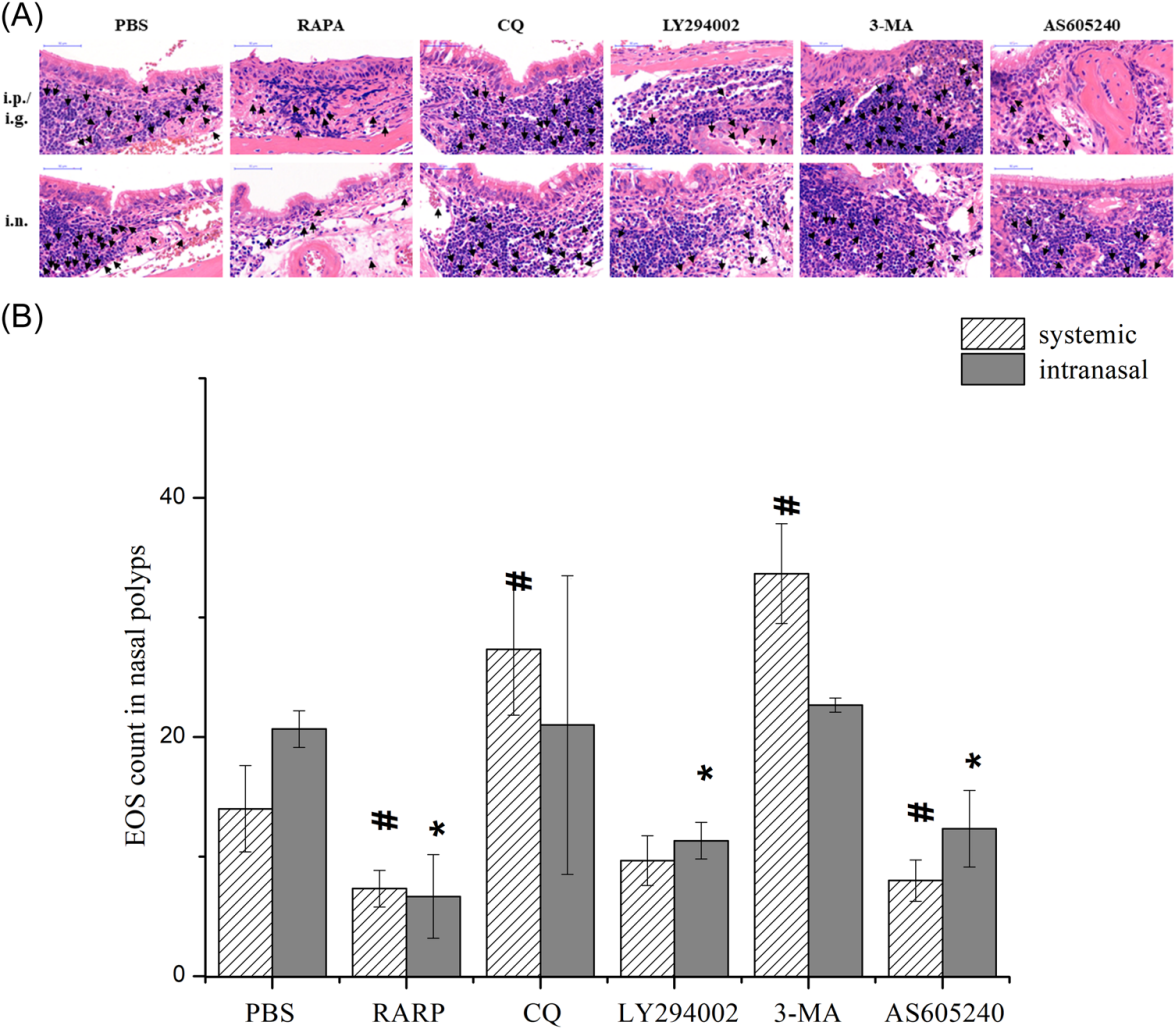
EOS count in nasal polyps detected by HE staining. (A) EOS infiltration in nasal polyps in each group. The black arrow indicates EOS. (B) Comparison of the number of EOS in nasal polyps in each group. ^#^ *p* < .05 vs. PBS‐i.p. *p < .05 vs. PBS‐i.n. EOS, eosinophil; HE, hematoxylin and eosin; PBS, phosphate buffered saline.

Hematoxylin and eosin staining revealed significantly reduced EOS infiltration in nasal polyps after systemic application of rapamycin and AS605240 compared with the control. Systemic application of CQ and 3‐MA significantly increased EOS infiltration in nasal polyps (Figure 7A,B).

Together, these results show that eosinophilic inflammation in eCRSwNP mice could be inhibited by promoting the autophagy; otherwise, eosinophilic inflammation could be promoted. Meanwhile, inhibition of the PI3K/Akt/mTOR pathway can further promote autophagy and inhibit eosinophilic inflammation.

### 3.4 ILC2s in eCRSwNP mice

**Figure 8:**
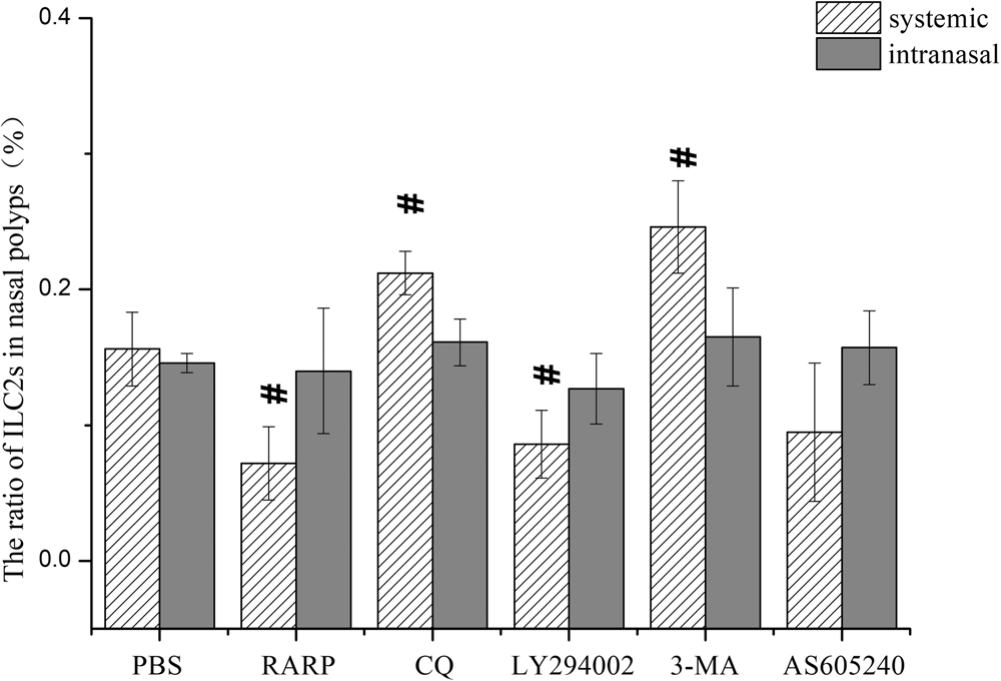
ILC2s ratio in nasal polyps detected by flow cytometry. ^#^ *p* < .05 vs. PBS‐i.p. *p < .05 vs. PBS‐i.n. PBS, phosphate buffered saline.

Flow cytometry showed that, compared with the PBS control group, ILC2s numbers in nasal polyps were reduced after systemic application of rapamycin and LY294002. By contrast, systemic application of CQ and 3‐MA increased the number of ILC2s in nasal polyps (Figure 8). These findings suggest that inhibiting the PI3K/Akt/mTOR pathway and promoting autophagy can reduce the number of ILC2s in the nasal polyps of eCRSwNP mice. Inhibition of autophagy can increase the number of ILC2s.

### 3.5 Tissue remodeling in eCRSwNP mice

**Figure 9:**
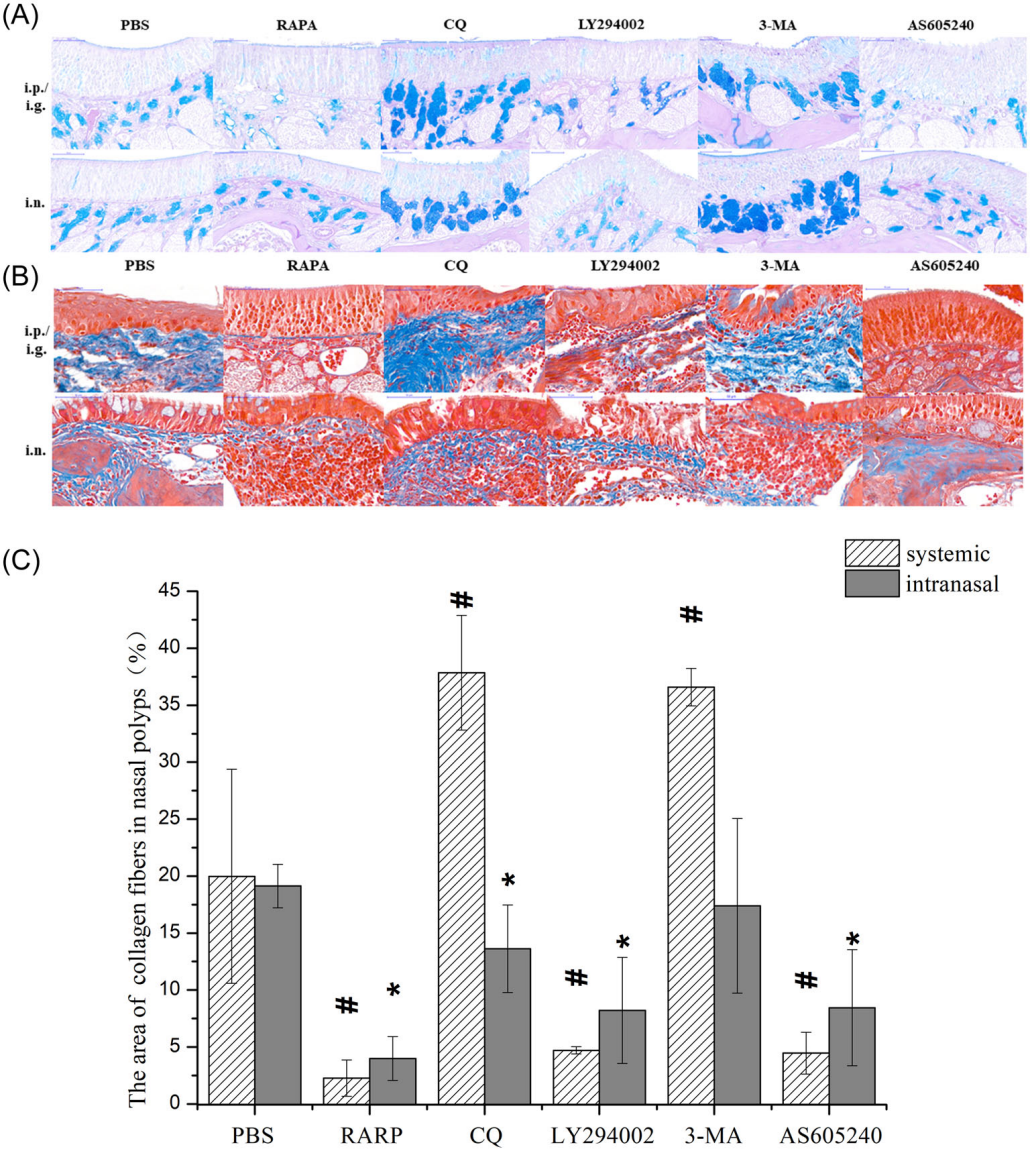
The degree of tissue remodeling in nasal polyps. (A) AB‐PAS staining was used to detect goblet cell proliferation in nasal polyps in each group. (B) MT staining was used to detect the collagen fiber proliferation in nasal polyps in each group. (C) The ratio of collagen fibers in nasal polyps of each group was compared. ^#^ *p* < .05 vs. PBS‐i.p. *p < .05 vs. PBS‐i.n. MT, Masson's trichrome; PBS, phosphate buffered saline.

Hematoxylin and eosin staining showed that there was no significant difference in the degree of epithelial cell injury in different groups. PAS‐AB staining showed that systemic and intranasal application of rapamycin, LY294002, and AS605240 reduced the number of goblet cells and the degree of glandular hyperplasia compared with the control. The number of goblet cells and the degree of glandular hyperplasia were increased after systemic and nasal administration of CQ and 3‐MA (Figure 9A and 9C). MT staining showed that systemic and intranasal application of rapamycin, LY294002, and AS605240 decreased the percentage of collagen fiber area compared with the control. Systemic and intranasal application of CQ and systemic application of 3‐MA significantly increased the percentage of collagen fiber area (Figure 9B,C). These results indicate that inhibiting the PI3K/Akt/mTOR pathway and promoting autophagy can attenuate the severity of tissue remodeling in eCRSwNP mice, whereas inhibiting autophagy can aggravate tissue remodeling.

### 3.6 Correlation among ILC2s, eosinophilic inflammation, and tissue remodeling

**Figure 10:**
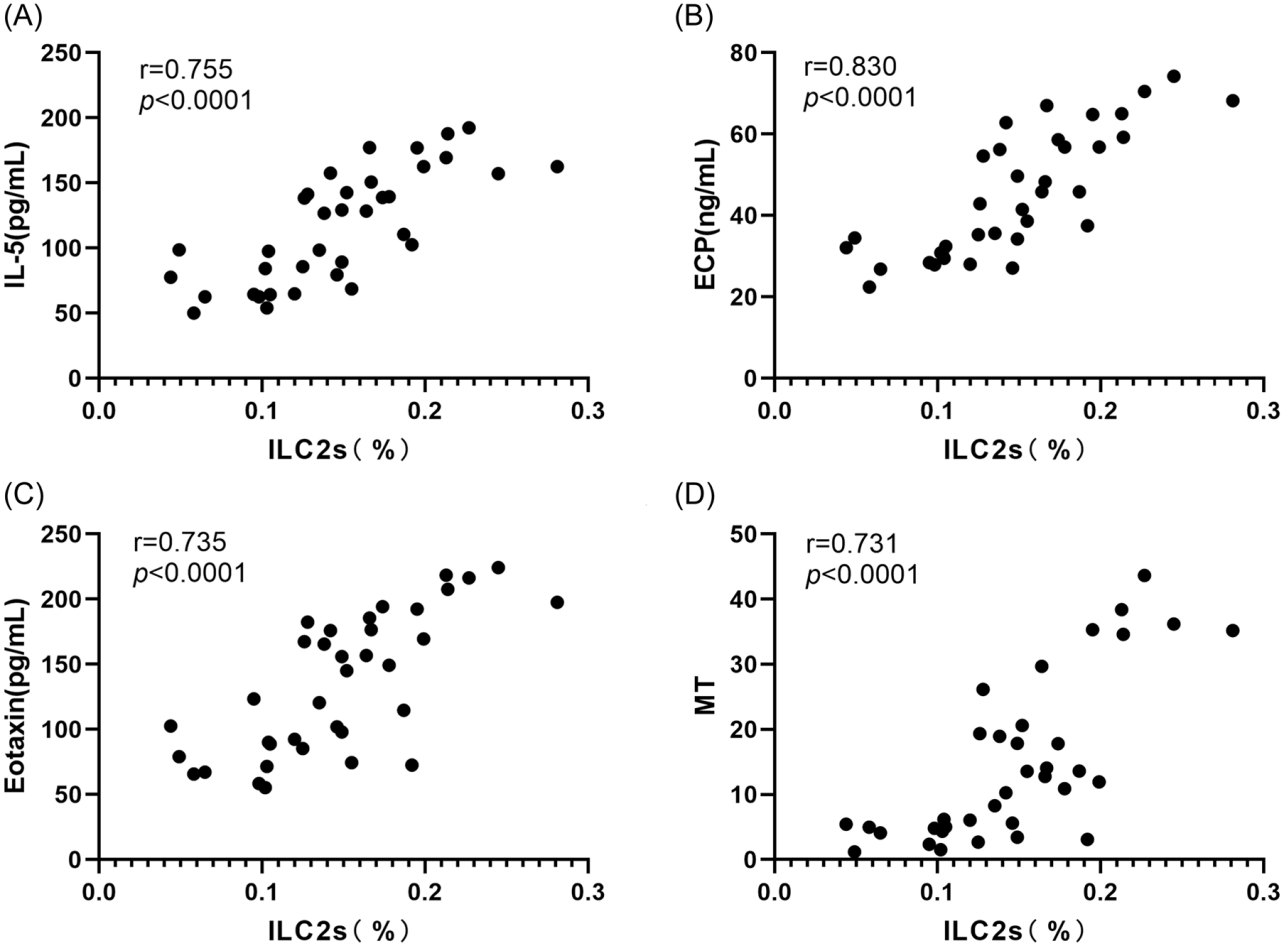
Correlation among ILC2s, eosinophilic inflammation, and tissue remodeling. (A) The correlation between ILC2s and IL‐5 in nasal polyps. (B) The correlation between ILC2s and ECP in nasal polyps. (C) The correlation between ILC2s and eotaxin in nasal polyps. (D) The correlation between ILC2s and MT staining results in nasal polyps. ECP, eosinophil cationic protein; MT, Masson's trichrome.

There was a strong positive correlation between ILC2s and IL‐5 in nasal polyps (*r* = .755, *p* < .001) (Figure 10A). ILC2s were strongly positively correlated with ECP (*r* = .830, *p* < .001) (Figure 10B) and eotaxin (*r* = .735, *p* < .001) (Figure 10C). There was also a strong positive correlation between ILC2s and MT staining results (*r* = .731, *p* < .001) (Figure 10D).

## 4 DISCUSSION

### 4.1 The main and novel findings emerging from this study

1. The PI3K/Akt/mTOR pathway plays roles in eosinophilic inflammation and tissue remodeling of eCRSwNP, in part by regulating the level of autophagy.

2. The downregulation of autophagy is a pathogenesis of eCRSwNP.

3. Autophagy can inhibit eosinophilic inflammation and tissue remodeling, in part by reducing the number of ILC2s.

The PI3K/Akt/mTOR pathway is closely related to eosinophilic inflammation and tissue remodeling. Inhibition of the PI3K/Akt/mTOR pathway can inhibit the chemotaxis and recruitment of EOS, reduce the survival time and response of EOS, and decrease the production of EOS‐related ribonuclease, thereby attenuating eosinophilic inflammation.^12^ In addition, inhibition of the PI3K/Akt/mTOR pathway can inhibit tissue remodeling in allergic airway inflammation.^13^ However, few studies have investigated the PI3K/Akt/mTOR pathway in nasal inflammation, although some have focused on hypoxia‐inducible factor‐1α, a downstream factor of this pathway.^14^ Inhibition of the PI3K/Akt pathway can decrease the production of inflammatory factors in CRSwNP, thereby attenuating inflammation.^15^ In eCRSwNP, IL‐33 can promote the production of IL‐4 and IL‐5 by activating the PI3K/Akt pathway.^16^

The PI3K/Akt/mTOR pathway also plays an important role in the regulation of autophagy. mTORC1 can phosphorylate autophagy‐related genes (ATG)13. The binding of hyperphosphorylated ATG13 to ATG1 is weakened, decreasing ATG1 kinase activity and inhibiting the downstream signal of autophagy. Vps34, a Class Ⅲ PI3K catalytic subunit, can catalyze the phosphatidyl inositol of membrane to form phosphatidylinositol 3 phosphate (PI3P). It can recruit molecules containing the PI3P‐binding domain to bind to the intima. It can also promote the binding of autophagy‐related proteins ATG21 and ATG24 to the membrane to form pre‐autophagy structures.^17^ One study showed that lipopolysaccharides can inhibit autophagy and promote the proliferation of fibroblasts in lung tissue by activating the PI3K/Akt/mTOR pathway.^18^ Rapamycin inhibits the PI3K/Akt/mTOR pathway, thereby inhibiting the differentiation of fibroblasts in nasal polyps and the production of collagen and extracellular matrix, and attenuating the occurrence of tissue remodeling.^19^ Chen et al.^20^ found that the Akt/mTOR pathway in nasal polyps was significantly activated and the level of autophagy decreased. Simsek et al.^21^ found that the PI3K/mTOR pathway was activated and LC3 expression decreased in nasal polyps.

In this study, we used OVA and *S. aureus* enterotoxin B to build a eCRSwNP mouse model, according to the previous literature.^10^ Other authors established eosinophilic mouse models with Aspergillus fumigatus antigens.^22^ In this study, we found that the broad‐spectrum PI3K inhibitor LY294002 and selective PI3Kγ inhibitor AS605240 had inhibitory effects on the PI3K/Akt/mTOR pathway. They significantly upregulated the level of autophagy in nasal polyps and reduced the severity of eosinophilic inflammation and tissue remodeling. Although the PI3Kγ inhibitor 3‐MA had an inhibitory effect on the PI3K/Akt/mTOR pathway, it also had an inhibitory effect on Vps34, generally reducing the level of autophagy and aggravating the degree of eosinophilic inflammation and tissue remodeling in nasal polyps. Therefore, we speculate that the PI3K/Akt/mTOR pathway might play roles in eosinophilic inflammation and tissue remodeling of eCRSwNP, in part by regulating the level of autophagy. The PI3K/Akt/mTOR pathway might also alter cytokine production, metabolism or immune cell activation in eCRSwNP. These will be elucidated in future studies.

Autophagy plays an important role in eosinophilic inflammation and tissue remodeling of the airway. MiRNA‐192‐5p can effectively reduce airway remodeling in asthma by inhibiting autophagy.^23^ However, the role of autophagy in CRS remains controversial. Qi et al.^24^ found that the levels of Beclin‐1, Beclin‐1 messenger RNA (mRNA), and LC3Ⅱ mRNA in nasal polyps decreased, but did not correlate with EOS infiltration. Choi et al.^6^ knocked out the ATG7 gene of bone marrow cells in an eosinophilic CRS mouse model, and found that autophagy defects promoted EOS infiltration, epithelial cell proliferation, and mucosal hypertrophy, and promoted the development of eosinophilic CRS. Finally, using IHC, WB, and TEM, Wang et al.^7^ showed that the level of autophagy in the nasal polyps of eCRSwNP patients was increased.

In this study, we found that the application of the autophagy activator rapamycin, and PI3K inhibitors LY294002 and AS605240 significantly increased the level of autophagy, inhibited eosinophilic inflammation, and reduced the severity of tissue remodeling in nasal polyps. The application of autophagy inhibitors CQ and 3‐MA reduced the level of autophagy and enhanced eosinophilic inflammation and tissue remodeling in nasal polyps. Tissue remodeling mainly manifested as the proliferation of goblet cells, glands, and collagen fibers. There was no difference in epithelial cell injury among the groups. In addition, CQ mainly inhibited autophagy by inhibiting the fusion of autophagosomes and lysosomes, and also inhibited LC3 degradation. Therefore, the application of CQ can lead to a significant increase in LC3 levels. These results are in accordance with some previous studies.^6^, ^24^ As a result, we speculate that the downregulation of autophagy is a pathogenesis of eCRSwNP. Thus, the recovery of normal autophagy levels might be a new target for eCRSwNP therapy.

ILCs are a newly discovered innate immune lymphocyte functionally similar to T helper (Th) cells. They can produce similar cytokines as Th cells but do not express antigen receptors.^25^ According to the production of cytokines and expression of transcription factors, ILCs can be divided into three subtypes: ILC1s, ILC2s, and ILC3s, which are functionally similar to Th1, Th2, and Th17 cells. ILC2s can promote eosinophilic inflammation by producing type 2 inflammatory factors. ILC2s also play a role in airway fibrosis and tissue remodeling by producing amphiregulin and promoting fibroblast activation.^26^ However, the role of ILC2s in CRSwNP remains controversial.^27^ This study found that the number of ILC2s in nasal polyps could be reduced by promoting autophagy and inhibition of the PI3K/Akt/mTOR pathway. By inhibiting autophagy and activation of the PI3K/Akt/mTOR pathway, ILC2s could be increased. In combination with the correlation among ILC2s, eosinophilic inflammation, and tissue remodeling indicators, we speculate that autophagy might inhibit eosinophilic inflammation and tissue remodeling, in part by reducing the number of ILC2s. The PI3K/Akt/mTOR pathway might upregulate the number of ILC2s to enhance the severity of inflammation in eCRSwNP. A previous study showed that autophagy and mitophagy of ILC2s play an important role in maintaining their survival and function,^28^ which does not completely agree with our study. This study mainly focused on the overall level of autophagy in tissues. We recommend that future studies focus on the autophagy of specific cells or selective autophagy, such as mitophagy, which could reveal the exact role of autophagy in eCRSwNP. However, the PI3K/Akt/mTOR pathway can also target T cells and nasal epithelial cells. The PI3K/Akt/mTOR pathway and autophagy are also important for T cells survival, metabolism, development and function.^29^, ^30^ These in eCRSwNP will be elucidated in future studies.

Meanwhile, the PI3K pathway and autophagy play important roles in maintaining normal physiological functions of the body. Systemic inhibition of the PI3K pathway and autophagy has significant side effects. This study explored nasal administration of drugs, which was not as effective as systemic administration but still showed some effects.

There were some limitations to this study. First, eCRSwNP mice were used in the control group, and each experimental group was compared with eCRSwNP. If a control group of mice could be sensitized and stimulated with PBS, it would be better. Second, the study mainly focused on the level of autophagy in whole tissue, and did not detect autophagy levels in specific cells such as ILC2s. In this study, we only detected the number of ILC2s in nasal polyps. The ILC2s culture in vitro and the survival, proinflammatory effector function and proliferation of ILC2s will be investigated in subsequent experiments. A ILC2s‐specific knock‐out mouse models should be applied to explore the exact role of ILC2s in next research.

## 5 CONCLUSIONS

In this study, the PI3K/Akt/mTOR pathway plays roles in eosinophilic inflammation and tissue remodeling of eCRSwNP, in part by regulating the level of autophagy. The downregulation of autophagy is a pathogenesis of eCRSwNP; therefore, the recovery of normal autophagy levels might be a new target for eCRSwNP therapy. Furthermore, for the first time, we speculate that autophagy can inhibit eosinophilic inflammation and tissue remodeling, in part by reducing the number of ILC2s.

## AUTHOR CONTRIBUTIONS

**Jin‐Jing Zhuo**: Data curation, formal analysis; writing—original draft. **Chen Wang**: Conceptualization, methodology. **Yi‐Long Kai**: Data curation, software. **Ying‐Ying Xu**: Formal analysis, methodology. **Ke‐Jia Cheng**: Writing—review & editing.

## CONFLICT OF INTEREST STATEMENT

The authors declare no conflicts of interest.

## ETHICS STATEMENT

All mice were managed in accordance with protocols approved by the Research Ethics Committee of the First Affiliated Hospital, College of Medicine, Zhejiang University (Zhejiang, China). All methods were carried out in accordance with relevant guidelines and regulations. All methods were reported in accordance with ARRIVE guidelines.

## Data Availability

The datasets used and/or analyzed during the current study are available from the corresponding author on reasonable request.
